# Haematologists as Genetic Counsellors for Haemoglobinopathies: Are They Prepared?

**DOI:** 10.3390/hematolrep17050048

**Published:** 2025-09-15

**Authors:** Michael Angastiniotis, Androulla Eleftheriou

**Affiliations:** Thalassaemia International Federation, 31 Ifigenias Street, 2007 Nicosia, Cyprus; thalassaemia@cytanet.com.cy

**Keywords:** hereditary anaemias, thalassaemia, genetic counselling

## Abstract

**Background/Objectives**: In haematology, a wide range of blood disorders are hereditary. The thalassaemias are hereditary anaemias characterised by a high burden of disease at the public health level, challenging the resources of many health systems. This review focuses on thalassaemias for which many countries have developed screening and prevention programmes. To manage this heavy burden, two approaches were introduced over the years. The first one focused on reducing the annual affected births consequent to appropriate non-directive genetic counselling, offering to the parents the chance to make an informed choice concerning their reproductive lives. The second approach was related to the development of curative treatments such as haematopoietic stem cell transplantation (HSCT) in the early years, with continued ongoing efforts for improvements, followed by successful advances in gene-based holistic cures in more recent years. Genetic counselling is a vital component in successful prevention, aiming at informing individuals who are found to be carriers and couples who are both carriers with a 25% risk at every pregnancy of having an affected child in the case of recessive, Mendelian inheritance. The issues are many, and that may have to be discussed, highlighting the level of skills which a genetic counsellor is expected to possess and utilise appropriately in every counselling session. The concern is that such trained and skilled professionals are few in number and not well integrated into the multidisciplinary groups addressing the control of these complex disorders. It is our experience that for blood disorders, counselling is rarely in the hands of qualified scientists. It is our firm belief that it is necessary to incorporate genetic counselling as an integral part of haematology services. **Methods**: To investigate current practices we have drawn on the experience of existing programmes, as well as published literature. **Results**: Currently, in almost all haemoglobinopathy prevention programmes, counselling is offered by the clinicians in charge of clinical care or, in some settings, by the nurse of the clinic or the screening laboratory scientist. **Conclusions**: The Thalassaemia International Federation suggests and is in the process of developing special training in counselling as part of haematology training, as well as professional development modules for those already in practice. Considering the complexity of the issues that must be discussed, a multidisciplinary approach to counselling should be considered where possible.

## 1. Introduction

In haematology, a wide range of blood disorders are hereditary. These include haemoglobin disorders (haemoglobinopathies), bleeding disorders, and rare anaemias. This review focuses on thalassaemia, for which many countries have developed screening and prevention programmes, albeit with limited effectiveness, as vital components of such programmes are absent in most cases. Several aspects of such programmes could also be applied to other haemoglobinopathies such as sickle cell disease or contribute a good basis for developing control programmes for a variety of other genetic, hereditary diseases.

Thalassaemias are inherited anaemias that place a significant burden on public health systems as well as patients and their families. This is attributed to their high prevalence across many populations worldwide and their lifelong requirement for disease-specific care, including a constant supply of blood for red cell transfusions, expensive medications, and meticulous ongoing monitoring with multidisciplinary care. They are caused by pathogenic variants in the globin genes of the haemoglobin molecule, resulting in chronic anaemia due to ineffective erythropoiesis and haemolysis, iron overload, and organ damage. Although disease severity varies, early and continuous clinical monitoring is essential to ensure both longevity and reduced comorbidity.

Haemoglobinopathies are the most common severe monogenic disorders globally, with approximately 7% of the global population being carriers of a pathological haemoglobin gene and over 500,000 affected children born each year, although these figures are believed by all involved to be a gross underestimation. This is consequent to the absence of national registries and updated and upgraded or new and well-designed epidemiological work, particularly in countries with large populations or high disease prevalence. The prevalence of thalassaemia is particularly high in the Middle East, Asia, and Mediterranean compared with Europe and North America, although migration patterns are changing the prevalence across regions [1,2]. Sickle cell disease shows the highest prevalence in Africa (~800/100,000), followed by the Middle East (~200/100,000) and India (~100/100,000), in contrast to Europe (~30/100,000), where population movements are also increasing the number of affected individuals [3].

In thalassaemia, chronic, regular transfusions aiming to reduce ineffective erythropoiesis and subsequent anaemia lead to iron overload and damage to vital organs. This can be prevented by daily iron chelation therapy, though it adds both clinical and financial burdens for patients and healthcare systems. Without strict adherence to monitoring and treatment, complication rates remain high, further increasing the disease’s overall burden [4,5].

To address this, two main strategies have emerged alongside improvements in clinical care. The first, introduced in the 1970s, focused on reducing the number of new affected births, aiming to prevent the patient population from growing and offer parents informed reproductive choices [6]. The second approach involved the development of curative treatments, with haematopoietic stem cell transplantation (HSCT) from HLA-compatible donors becoming available in the 1980s [7]. More recently, gene-based therapies—such as gene addition and gene editing—have shown promising results [8]. In parallel, new pharmacological treatments are emerging to reduce the burden of anaemia and transfusion requirements through medications such as luspatercept (promoting erythroid differentiation) [9] and mitapivat (managing red cell health by activating pyruvate kinase) [10]. However, the high cost of these innovations remains prohibitive for many patients in low- and middle-income countries, widening global health disparities.

All these issues should be familiar to treating physicians—typically paediatric or adult haematologists—though primary care providers, paediatricians, and internists may also be involved. Haematologists should also be heavily involved in national prevention programmes, given their expertise in laboratory diagnosis as well as other aspects of disease control.

Prevention programmes are typically coordinated at the national level and involve raising public awareness, screening to identify asymptomatic carriers (ideally prior to conception), and providing genetic counselling, followed by possible interventions such as preimplantation genetic testing or prenatal diagnosis, according to the informed choice of at-risk individual or couples [6]. Such measures are of vital importance to the wellbeing not only of the potential offspring but also for supporting carrier couples, who may face painful decisions about their future and relationships.

Currently, we are aware of 25 countries [2] where some effort at preventing or reducing thalassaemia births is ongoing. In a minority of countries, these are complete national policies including all facilities and offering choices such as prenatal diagnosis or pre-implantation genetic testing. In many others, the programme stops at screening for premarital couples or in antenatal clinics. In all cases, however, genetic counselling is or should be offered to individuals or at-risk couples.

## 2. Methodology

Understanding who provides counselling in countries where haemoglobin disorders are prevalent, whether they are qualified through special training, and what is known about counselling outcomes is important. Our sources of information include the following:

- Experience from the Cyprus thalassaemia programme: It was evident from the early stages of the programme that children were being born with severe thalassaemia syndromes, because the parents were either not informed (49% of 55 new affected births) or misinformed (13% of the 55) [6]. While post-screening counselling was part of the service, the responsible personnel were not initially defined. These negative outcomes resulted in a centralised counselling service based on the doctors of the thalassaemia centre in Nicosia, which is the hub of the national programme.
- International experience from the Thalassaemia International Federation (TIF): The TIF maintains communication with its member organisations across 64 countries along with the treating physicians in these countries, who are mainly paediatricians and haematologists. While most of these countries have screening or diagnostic facilities, systematic national prevention with screening, counselling, and availability of prenatal foetal testing was recorded in only 25 countries [2].
- Literature review: We conducted a targeted search through Pubmed and Google Scholar using keywords such as genetic counselling thalassaemia and sickle cell disease or anaemia for English language articles published after 2000. The objective was to identify articles that pose the question of who provides counselling in these blood disorders for “at-risk” couples. Our aim was to identify studies discussing who provides counselling for “at-risk” couples with these blood disorders. Articles addressing general population awareness or using the term “genetic counselling” solely in reference to patient or caregiver counselling were excluded. The following articles were considered indicative of our objectives. (see Table 1)

## 3. Counselling

Genetic counselling is a crucial component of prevention, aiming to inform individuals identified as carriers and couples who are both carriers, in the case of recessive inheritance, of the possible consequences for their own health and for their offspring. In the case of haemoglobinopathies, the vast majority of carriers are asymptomatic and can be detected through haematological testing, often supplemented by molecular analysis. If both partners are carriers, then in the recessive mode of inheritance, each pregnancy carries a 25% risk of producing a child affected by a syndrome, which may range from severe to relatively mild depending on the specific genes and genetic modifiers involved.

The options available for carrier couples are summarised in Table 2 and often involve major decisions with serious emotional and social consequences. Decisions are influenced by cultural norms, religious beliefs, legal frameworks, literacy levels, and personal preferences. Similarly, policymakers give emphasis or direct the goals of prevention according to these same cultural influences. For instance, avoiding “incompatible marriage” is the emphasis of some national programmes [18]. Consanguineous marriage is a preferred practice in many countries which may influence birth incidence and the prevention programme [19]. Other cultures offer solutions like prenatal diagnosis and termination of affected pregnancies [20] as well as pre-implantation genetic testing [21].

Regardless of cultural context, genetic counselling is an essential process that is necessary to offer knowledge by which affected people can make informed choices regarding their relationships and reproductive lives. It is a personalised service that addresses the needs of each individual or couple in assessing the risk of having an affected child. Ensuring that counselling is a priority in all prevention programmes, with recognition of the specialised expertise required, is of paramount importance [23,24,25].

## 4. Who Provides Counselling?

Ideally, genetic counselling is provided by trained medical professionals known as genetic counsellors [26,27]. These specialists have training in medical genetics and diagnostics, as well as the communication skills needed to inform and support individuals and families who may receive potentially distressing information from genetic testing. Counsellors help clients process this information and make informed decisions about actions, outlined in Table 2, that can have a significant impact on personal and social implications.

Some of the issues which the counsellor needs to address include the following:

- Consent for pre-symptomatic testing: This follows information about the reasons for testing and is satisfied to a highly variable extent by an ongoing public awareness campaign. This again depends on issues such as literacy and the effectiveness of measures reaching the public, such as using the media, flyers, lectures, or school teaching.
- Interpretation of laboratory results: This means knowledge of the screening algorithms and the laboratory tests performed, as well as knowledge of the mutations involved and genotype or phenotype possibilities. Sometimes it requires teamwork to reach a correct diagnosis, and thus an individual counsellor must ensure that he or she has the correct final diagnosis to provide advice; otherwise, if there is uncertainty, then this should be expressed and explained.
- Carrier status explanation: Counsellors explain what being a carrier (heterozygote) means for the individual’s health and future prognosis.
- Family and medical history analysis: This includes assessment of pedigrees to interpret inheritance patterns.
- Screening type-related issues:
  a. Premarital screening: Decisions about marriage and possible separation can be a painful dilemma for individuals or couples.
  b. Antenatal clinic screening: Here, the woman is found to be a carrier with an ongoing pregnancy, and she is faced with the task of bringing her partner in for testing, with the possible need for prenatal diagnosis and finally the question of termination of the pregnancy if the foetus is found to be affected.
  c. Family members: This involves testing of other family members, especially where consanguinity is common. Is there an issue of disclosure of the presence of asymptomatic carriers in unsuspecting relatives? Does the proband want them to know, or do they want to be screened? The right not to know must also be respected.
  d. Non-paternity: Counsellors may encounter issues related to non-paternity and must handle such matters sensitively.
- Risk assessment: This means estimating the likelihood of disease occurrence or recurrence.
- Education: Information is provided on the natural history of the condition, inheritance pattern, diagnosis, management, and prevention.
- Phenotypic variability: The affected offspring may be transfusion-dependent with a severe syndrome but may also have a milder non-transfusion-dependent syndrome which, later in life, may bring about complications and increased morbidity. This may aggravate the dilemma for parents, especially if there is a degree of uncertainty.
- Socioeconomic implications: Counsellors discuss potential effects on employment, insurance, and other aspects of family life in certain settings.
- Advances in treatment: Individuals and couples are informed about current and emerging therapies, which may alter prognoses and influence reproductive decisions.

## 5. Quality of Counselling

The quality of genetic counselling depends on several key factors, apart from the key component of the extent to which counsellors stay updated on new developments in all aspects of care:

1. Communication skills: Effective counselling requires active listening, patience, empathy, privacy, and the ability to build trust with individuals and couples.
2. Ethical principles: Counsellors must uphold confidentiality and a non-directive approach supporting informed choice. They should understand the psychosocial and family factors that can influence choices. The autonomy of the couple must be respected. Some couples faced with difficult choices suggest or request that the counsellor should decide or direct a decision that should be theirs. (“What would you do doctor?”) The counsellor must support the couple in the reasoning and decision-making process while absolutely avoiding expressing an opinion.
3. Disclosure to family members: In communities where consanguinity is common, disclosing carrier status to relatives may help identify carriers. Privacy laws may prohibit disclosure of information, and thus the choice to disclose rests on the carrier and not the counsellor, unless consent is obtained.
4. Cultural considerations: Counsellors should be sensitive to the religious and cultural influences that may affect a couple’s choice. This requires knowledge of diverse cultures and the ability to explore the couple’s beliefs without prejudice. Avoiding being critical of prejudices is important. Such considerations are frequent in multi-cultural societies and are increasing following the migration flows of recent years.
5. Language: Communication may be hindered when language barriers exist. Translators may be needed—though ideally not relatives—to maintain confidentiality and ensure unbiased decision making.
6. Repetition and reinforcement: Complex genetic concepts may be difficult to grasp during the first session, particularly when emotional tension is high. Repeat sessions may be necessary to ensure understanding.
7. Informed choice: Decisions should be based on accurate, non-directive information, considering risk assessment, family goals, and cultural, ethical, and religious values, as well as emotional, familial, and social factors.

When discussing reproductive choices (Table 2), the following principles apply:

- Questions about choosing a partner or separating or not are for the individual or couple to decide free from external influence.
- Prenatal diagnosis is optional; methods, risks, and potential complications should be fully explained. Decisions regarding pregnancy termination, if relevant, must remain voluntary.
- Pre-implantation genetic diagnosis, including associated risks, costs, and religious acceptability, must be discussed. The fate of stored embryos must also be considered.
- Intrauterine treatment of hydrops can be offered, with counselling about postnatal transfusion dependence.

These discussions are inherently complex and demand a high level of skill from the genetic counsellor. A key concern is that professionals with this specialised expertise remain limited in number.

## 6. Availability of Skilled Counsellors

According to the relatively recent literature, the global number of certified genetic counsellors in 2018 was approximately 7000, practicing in at least 28 countries [28]. Even if this number has doubled in the last 7 years, the number of qualified genetic counsellors remains insufficient to meet the demand. Considering an estimated 500,000 haemoglobinopathy-affected births annually, roughly 1 million individual parents would theoretically require counselling each year. It is therefore clear that counselling is rarely provided by qualified genetic counsellors.

This raises concerns about the quality of counselling offered by non-specialists [29]. Studies comparing the outcomes of counselling by certified counsellors versus clinicians have reported variable results. For example, Rowley et al. in 1995 [30] used indicators such as (1) the proportion of β-thalassaemia births after counselling, (2) the knowledge of at-risk partners post-counselling, and (3) whether the counselled individual brought their partner for testing. The only significant difference between groups was in partner testing. The authors concluded that clinicians and nurses, following brief training, could provide genetic counselling for haemoglobinopathy carriers as effectively as professional counsellors, provided that the training was adequate.

Other outcome measures for genetic counselling include increasing understanding, enhancing coping and adaptation, facilitating informed choice, and mitigating health risks [31]. Moreover, recent advances in genomics, potential curative therapies, and emerging pharmacological treatments require counsellors to be well informed [32]. In this regard, practising haematologists may be well positioned to inform patients.

Moreover, the increased attention to the impact of lifestyle, environment, and other influences on health in the context of “precision medicine” make the art and science of genetic counselling even more demanding of specialised knowledge and understanding of all factors that may influence wellbeing and health. A further complication is the increasing cultural and linguistic mix in many populations due to migrations, which require the counsellor to be aware of cultural considerations as well as social determinants which can influence outcomes. Additionally, counsellors must possess psychosocial skills to provide effective support.

In recent years, the National Society of Genetic Counsellors (NSGC, USA) proposed standards for the “critical assessment of quality” and for the reporting of genetic counselling as a “complex intervention”, as well as evaluating genetic counselling outcomes. The resulting checklist includes 23 items classified within eight domains (not all applicable in specific services) [33]. The checklist is an indicator of the complex nature of genetic counselling, as illustrated in Table 3.

This section can be divided into subheadings to provide a concise and structured description of the findings, their interpretation, and the conclusions that can be drawn.

## 7. The Haematologist as a Counsellor

Of particular relevance to this paper is the fourth domain of the NSGC standards (Table 3), which addresses the provider and their qualifications. We assume that fully trained and qualified genetic counsellors are not available for the majority of haemoglobinopathy programmes across the globe. While several universities outside the USA and Europe (including India, with a huge haemoglobinopathy population) are now offering courses in genetic counselling, it will take time before a substantial number of qualified counsellors are available.

Currently, counselling in most haemoglobinopathy prevention programmes is offered by the clinicians in charge of clinical care, laboratory scientists responsible for screening, and nurses. In some situations, antenatal screening of pregnant women is practiced, and thus the obstetric team members act as counsellors.

In these scenarios, a question arises: is the quality of counselling assured? Many healthcare professionals may rely primarily on their understanding of Mendelian inheritance for risk assessment without addressing the broader complexities of genetic counselling. Yet, genetic counselling is an integral part of both patient care and prevention programmes, which are core components of haematology. For example, is a haematologist ready to discuss the implications of consanguineous marriage with an at-risk couple? The haematologist may be aware of the various mutations on the relevant genes, but the phenotype resulting from various combinations must also be known before advising a couple.

A haematologist may be best positioned to explain curative options, but they must also communicate the advantages and limitations of each approach without overoptimism, which may affect a couple’s choices. Providing emotional support requires time to listen to concerns, a resource clinicians may lack. Communication skills are variable among clinicians but also fundamental in any counselling session. Supporting patients amid uncertainty is particularly challenging, since this can increase patient anxiety.

The haematologist acting as a counsellor must also be aware of bioethical considerations and the legal frameworks which govern all issues that might arise, including termination of pregnancy. In addition, for those working in multinational societies where various cultures and religions coexist, cultural competence is another qualification to be added to the skills of the counsellor [34,35]. Sensitivity to cultural attitudes is essential in understanding decisions and maintaining a non-directive attitude. Couples in some cultures, influenced by religion, are expected to separate if there is a genetic risk; in other cultures, the emotional attachment dictates staying together, and thus a prenatal diagnosis is more easily accepted.

Finally, the haematologist is often the physician who continues to see the couple after they have made an informed decision, whether they choose prenatal diagnosis or pre-implantation testing. The counsellor’s role extends beyond the initial screening session, providing ongoing support when outcomes are challenging, such as when deciding on termination of an affected pregnancy or awaiting the result of an implanted embryo. Support does not end with post-screening sessions; it is a continuous process that ensures the couple feels guided and understood throughout.

## 8. Conclusions

This manuscript does not suggest that haematologists are inherently incapable of providing genetic counselling. While other scientific or medical specialties may also contribute to counselling, the focus of this paper remained on the primary responsibility and role of haematologists in this field. However, for most, prior and ongoing training—encompassing updated knowledge on both existing practices and new advances—is essential, a requirement that has been rarely emphasized in the past or recent literature. Given the complexity of the service, another possible solution is that genetic counselling of these disorders is provided by more than one practitioner, namely either a qualified counsellor in collaboration with a clinician or a clinician with a psychologist, social worker, or even a laboratory scientist. Such combinations will depend on the skills and knowledge of the various members of the team. They may work together so that common decisions may be reached or have separate interviews with a final session to discuss the individual’s or couple’s impressions and decisions.

Training or retraining programmes should be designed for healthcare professionals, whether haematologists or nurses, or other groups to ensure that standards are reached and “customers” benefit from counselling sessions so that they can make truly informed choices. Teaching techniques such as small group discussions and role play and stimulation can go a long way to increasing the understanding of what counselling entails, strengthening experience [36]. If such training is not part of undergraduate or postgraduate curricula, professional societies should promote specialised courses and professional development modules.

Genetic counselling must be incorporated as an integral component of haematology services, with one or more members of this specialty acquiring the knowledge and skills required for comprehensive counselling. The specifics of who provides the training and for how long are organisational questions, although collaboration with clinical genetics services or academic departments is an important step.

To provide answers to the many questions that this topic gives rise to, further research is needed. Currently, most research is conducted in countries where postgraduate genetic counselling practitioners are already available and involved in haematological counselling.

The Thalassaemia International Federation recommends the following:

1. Genetic counselling is a complex service that haematologists are called upon to fulfil as part of their duties.
2. Specialised training is recommended as part of haematology education, considering the complexity of the issues that must be addressed.
3. A multidisciplinary approach to counselling should be considered whenever possible.
4. Professional development modules for practicing haematologists involved in counselling should be adopted to maintain high standards and quality as part of ongoing maintenance of qualifications.

## Figures and Tables

**Table 1:** Selection of articles discussing counselling for haemoglobin disorders.

Country	Reference	Year	Comment
Thailand	Dhamcharee, V. et al. [11]	2001	Addresses the issue as to who provides counselling as well as the difficulties when administrative support is lacking.
Ghana	Anie, K.A. et al. [12]	2016	Discusses the development and implementation of a sickle cell counsellor training and certification program.
India	Muthuswamy, V. [13]	2011	Concludes that a primary care physician, paediatrician, or “many times” an obstetrician is the ideal clinician who need to undergo training in genetic counselling.
Saudi Arabia	Alswaidi, F.M. et al. [14]	2006	Due to the limited number of qualified genetic counsellors in Saudi Arabia, the majority of counselling clinics are run by paediatricians.
Malaysia	Ngim, C.F. et al. [15]	2013	Genetic counselling for thalassemia carriers is conducted by non-geneticist health care workers in many countries. The aim of the study was to assess Malaysian health care workers’ genetic counselling practices.
China, Hong Kong, SE Asia consultation	Zayts, O. et al. [16]	2013	In HK, counselling services are provided by a range of professionals, including clinical geneticists, obstetricians, nurses, oncologists, surgeons, and pathologists. The authors suggest research into current practices to inform about the curricula for in-service training.
Indonesia	Setiawan, H. et al. [17]	2022	Knowledge and competence of nurses in providing genetic counselling interventions in Indonesia needs to be improved through a coaching clinic. They suggest a “coaching clinic”, using learning methods such as discovery learning, small group discussion, role play, and simulations.

**Table 2:** Choices available for couples “at risk”. Source: Eleftheriou, A. and Angastiniotis, M. The Global Thalassaemia Review 2024. Thalassemia International Federation; 2024 [22].

Risk Identified	Choices
Before marriage or pregnancy	To avoid marrying another carrier; To separate from a relationship that puts their future children “at risk”; To marry their chosen partner, with knowledge of the risk involved.
After marriage or cohabitation	To proceed with a pregnancy, accepting the risk of possibly bearing an affected child; To avoid having children (e.g., choosing adoption); To undergo prenatal diagnosis, choosing to either accept an affected child or pregnancy interruption; To use pre-implantation genetic diagnosis as an alternative to prenatal diagnosis and thus avoid pregnancy interruption.
When already pregnant	To undergo prenatal diagnosis (if in early pregnancy); To accept any outcome with no further action; To interrupt the current pregnancy with no further action.

**Table 3:** Standards for the reporting of genetic counselling interventions in research and other studies (GCIRS). Modified from: Hooker, G.W. et al. [33].

Domain	Examples of the Items to be Considered
Indication for genetic counselling	The reason for genetic counselling Whether the counselee is asymptomatic or symptomatic
Other components	Genetic testing before, after, or at the time of counselling Other clinician interactions
Intervention delivery	Delivery mode (telephone, in person, telemedicine, couple, or single) Physical setting (hospital, clinic, public, or private)
Provider of counselling	Qualifications Number of healthcare professional involved
Risk content and communication	Basis of risk assessment (family history, test results, and personal history) Type of risk information provided (frequencies and absolute or relative risk)
Educational content	Educational tools (booklets, flyers, web applications, etc.) Educational models or theories applied
Psychotherapeutic content	Goals (decision making, family communication, and facilitate coping and adaptation) Psychotherapeutic models or theories
Duration	Length of the genetic counselling interaction Number of genetic counselling interactions (including follow-up sessions)

## Data Availability

The original contributions presented in this study are included in the article. Further inquiries can be directed to the corresponding author(s).
